# CGKB: an annotation knowledge base for cowpea (*Vigna unguiculata L*.) methylation filtered genomic genespace sequences

**DOI:** 10.1186/1471-2105-8-129

**Published:** 2007-04-19

**Authors:** Xianfeng Chen, Thomas W Laudeman, Paul J Rushton, Thomas A Spraggins, Michael P Timko

**Affiliations:** 1 Department of Microbiology, University of Virginia Health System, Charlottesville, VA 29908, USA; 2 Academic Computing Health Sciences, Information Technology and Communication, University of Virginia, Charlottesville, VA 22908, USA; 3 Department of Biology, University of Virginia, Charlottesville, VA 22904, USA

## Abstract

**Background:**

Cowpea [*Vigna unguiculata *(L.) Walp.] is one of the most important food and forage legumes in the semi-arid tropics because of its ability to tolerate drought and grow on poor soils. It is cultivated mostly by poor farmers in developing countries, with 80% of production taking place in the dry savannah of tropical West and Central Africa. Cowpea is largely an underexploited crop with relatively little genomic information available for use in applied plant breeding. The goal of the Cowpea Genomics Initiative (CGI), funded by the Kirkhouse Trust, a UK-based charitable organization, is to leverage modern molecular genetic tools for gene discovery and cowpea improvement. One aspect of the initiative is the sequencing of the gene-rich region of the cowpea genome (termed the genespace) recovered using methylation filtration technology and providing annotation and analysis of the sequence data.

**Description:**

CGKB, Cowpea Genespace/Genomics Knowledge Base, is an annotation knowledge base developed under the CGI. The database is based on information derived from 298,848 cowpea genespace sequences (GSS) isolated by methylation filtering of genomic DNA. The CGKB consists of three knowledge bases: GSS annotation and comparative genomics knowledge base, GSS enzyme and metabolic pathway knowledge base, and GSS simple sequence repeats (SSRs) knowledge base for molecular marker discovery. A homology-based approach was applied for annotations of the GSS, mainly using BLASTX against four public FASTA formatted protein databases (NCBI GenBank Proteins, UniProtKB-Swiss-Prot, UniprotKB-PIR (Protein Information Resource), and UniProtKB-TrEMBL). Comparative genome analysis was done by BLASTX searches of the cowpea GSS against four plant proteomes from *Arabidopsis thaliana, Oryza sativa, Medicago truncatula*, and *Populus trichocarpa*. The possible exons and introns on each cowpea GSS were predicted using the HMM-based Genscan gene predication program and the potential domains on annotated GSS were analyzed using the HMMER package against the Pfam database. The annotated GSS were also assigned with Gene Ontology annotation terms and integrated with 228 curated plant metabolic pathways from the *Arabidopsis *Information Resource (TAIR) knowledge base. The UniProtKB-Swiss-Prot ENZYME database was used to assign putative enzymatic function to each GSS. Each GSS was also analyzed with the Tandem Repeat Finder (TRF) program in order to identify potential SSRs for molecular marker discovery. The raw sequence data, processed annotation, and SSR results were stored in relational tables designed in key-value pair fashion using a PostgreSQL relational database management system. The biological knowledge derived from the sequence data and processed results are represented as views or materialized views in the relational database management system. All materialized views are indexed for quick data access and retrieval. Data processing and analysis pipelines were implemented using the Perl programming language. The web interface was implemented in JavaScript and Perl CGI running on an Apache web server. The CPU intensive data processing and analysis pipelines were run on a computer cluster of more than 30 dual-processor Apple XServes. A job management system called Vela was created as a robust way to submit large numbers of jobs to the Portable Batch System (PBS).

**Conclusion:**

CGKB is an integrated and annotated resource for cowpea GSS with features of homology-based and HMM-based annotations, enzyme and pathway annotations, GO term annotation, toolkits, and a large number of other facilities to perform complex queries. The cowpea GSS, chloroplast sequences, mitochondrial sequences, retroelements, and SSR sequences are available as FASTA formatted files and downloadable at CGKB. This database and web interface are publicly accessible at .

## Background

Cowpea [*Vigna unguiculata *(L.) Walp.] is one of the most important food and forage legumes in the semi-arid tropics and a valuable and dependable commodity for farmers and grain traders with ~21 million acres grown worldwide and an annual production of over 3 million tons [1,2]. It is grown mostly by poor farmers in developing countries, with 80% of production taking place in the dry savannah of tropical West and Central Africa [2]. Despite its importance, cowpea has received relatively little attention from a research standpoint and remains to a large extent an underexploited crop where relatively large genetic gains can be made with only modest investments in both applied plant breeding and molecular genetics.

Cowpea growth and yield are constrained by a variety of biotic and abiotic factors. Insects, fungi, bacteria, parasitic plants and nematodes are the major biotic stresses, and drought, salinity and heat are among the major environmental limitations to cowpea productivity [1,2]. One of the major goals of cowpea breeding and improvement programs is to combine resistances to numerous pests and diseases and other desirable agronomic traits, such as those governing maturity, photoperiod sensitivity, plant type, and seed quality. New opportunities for improving cowpea exist by leveraging the emerging genomic tools and knowledge gained through research on other major legume crops and model species.

The size of the cowpea nuclear genome has been estimated at 620 megabases (Mb), making it one of the smaller genomes present in leguminous plants as well as among vascular plants [3]. It is well documented that vascular plant genomes contain significant amounts of heavily methylated (hypermethylated) repetitive DNA surrounding by less methylated gene-rich (hypomethylated) regions [4-7]. Cytosine methylation content is positively correlated with genome size and complexity with 5-methyl-cytosine (5 mC) content ranging from 5% to 25% of total cytosine, depending on the species. Whereas the bulk of DNA methylation in mammals is confined to the short symmetrical sequence 5'-CG-3', DNA methylation in plant genomes is found in three nucleotide-sequence contexts: CG and two categories of non-CG sites: symmetrical CNG and asymmetric CHH sites (where N is any nucleotide and H is A, C or T) [8-10]. At the chromosomal level, an analysis of cytosine methylation levels in Arabidopsis chromosomes showed that there is a gradual increase of methylation along the genomic region analyzed: CpG methylation in the euchromatic fraction, CpG and CpNpG methylation at the euchromatin/heterochromatin transition and an additional asymmetrical methylation in the repeated-heterochromatic fraction. The density of DNA 5-methylcytosine methylation increased from the euchromatin towards the heterochromatin. The most methylated repeated family at CpG, CpNpG and asymmetrical sites is the 5S ribosomal DNA, highly methylated even though it is transcribed [11].

Methylation filtering (MF) allows for the selective cloning of hypomethylated regions of the plant nuclear genome [12]. MF has been successfully applied to the shotgun sequencing of the genomes of several plant species, allowing an examination of the content of the gene-rich regions referred to as the genespace [12-16]. A pilot study was carried out to determine whether GeneThresher^® ^methylation filtering technology [17] could be positively applied to analyzing the genespace of cowpea. Both methylation filtered (MF, GeneThresher^® ^technology) and unfiltered (UF) libraries were constructed, clones were picked at random from both libraries and the insert sequences determined and analyzed to estimate filtering power. The gene enrichment achieved by GeneThresher was determined by comparing the rate of gene discovery between MF and UF sequences. Detection of genes was accomplished by an NCBI-BLASTX search (parameters: -e 0.01; -b 5; -v 5) of the curated Arabidopsis protein database [13,17]. The results of this pilot study showed that the GeneThresher^® ^technology produced a 4.1-fold enrichment of gene-rich clones from cowpea genomic DNA libraries and estimated the size of the hypomethylated, gene-rich space of cowpea to be approximately 151 Mb. Using empirically derived results from the Orion Sorghum GeneThresher project and a simulation conducted on finished Arabidopsis sequence [13], we estimated that in order to sequence tag some portion of ~95% of the genes in the cowpea genome, we would need to generate ~252,000 GeneThresher sequences assuming an average read length of 600 bp and a 151 Mb genespace. This 1 × of raw sequence would encompass ~67% of the predicted genespace (Timko, M.P., manuscript in preparation).

**Figure 1:**
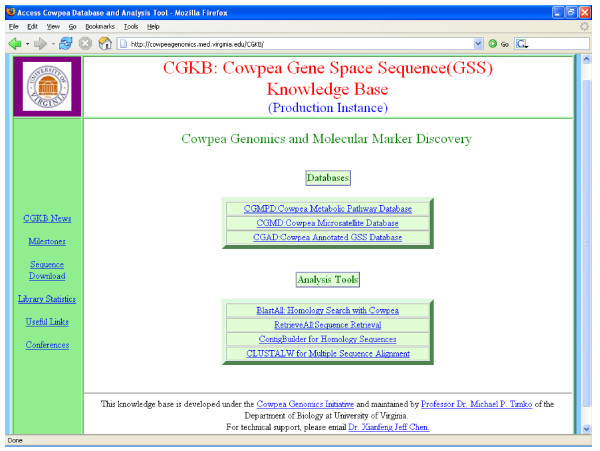
Screenshot of the cowpea genespace knowledge base.

The Cowpea Genomics Initiative (CGI) funded by the Kirkhouse Trust, a UK-based charity [18], undertook the goal of sequencing the cowpea genespace and providing annotation and analysis of the sequence data. The Cowpea Genespace/Genomics Knowledge Base (CGKB) was created at UVa to manage, analyze, and disseminate information derived from the sequencing of cowpea GSS. Currently no database of genomic resources exists for the cowpea research community. Therefore, a public genomics database for cowpea-centric information that allows users to acquire and access genomic sequence data interactively, analyze data, and generate reports in a user-friendly, web-accessible, and graphical manner is a key public service. Such an integrated resource will also benefit legume researchers by making comparative legume genetic and genomic information available to all other pan-legume researchers via a web-based interface. This integrated database will provide a platform for integrated data analysis and pan-legume data mining. Thus, CGKB has been developed under this initiative and is an integrated and annotated resource with features of both homology-based and HMM-based annotations, enzyme and pathway annotation, GO term annotation, toolkits, and a large number of other facilities to perform complex queries. We used JavaScript and Perl/CGI for most of the front end applications. The database is found at  (see figure 1) and is open to the public.

## Construction and content

### Data processing and analysis

The primary sequence dataset for the CGKB consists of a total of 298,848 GSS isolated by methylation filtering of the cowpea genomic DNA. The FASTA formatted cowpea sequence files generated using Phred basecalling were vector trimmed. Contaminant sequences, defined as sequences that at the time of initial annotation are believed to be derived from vector, microbial, fungal (yeast), viral or animal genomes, were removed. Chloroplast, mitochondrial and transposon/retrotransposon DNA sequences were also removed. Choloroplast, mitochondrial and transponson/retrotransposon sequences were determined by BLAST significant similarity scores equal to or less than 1 × 10^-10 ^when compared to relevant public databases. A total of 9985 chloroplast sequences, 856 mitochondrial sequences and 2608 transponson/retrotransposon-like sequences were identified in the dataset and these sequences are downloadable from the CGKB website. The remaining 263,425 MF nuclear sequences with average length of 610 bp were subjected to the annotation and data analysis processing pipeline. We used the Perl programming language [19] to implement data processing and analysis pipelines incorporated with various analysis algorithms. Both BLAST and Hidden Markov Model (HMM)-based algorithms are CPU intensive for genome scale data analysis. These CPU intensive data analysis pipelines were run on a computer cluster of over 30 dual-processor Apple XServes. A job management system called Vela was also created as a robust way to submit and manage large numbers of running data analysis jobs to the Portable Batch System (PBS) [20] in our distributed Apple OSX-based computer cluster.

### Comparative plant genome analysis

The complete sequence is currently available for two vascular plant genomes, *Arabidopsis thaliana *[21] and *Oryza sativa *[22]. An international effort is also underway to sequence *Medicago truncatula *[23] as the nodal species for comparative and functional legume genomics. In addition, the draft genome of the woody angiosperm *Populus trichocarpa *(Torr. & Gray) (black cottonwood) has been completed and is available for comparison [24]. We performed comparative genome analysis using these four plant genomes. Each cowpea GSS was searched with BLASTX against proteomes of *Medicago truncatula, Arabidopsis thaliana*, *Oryza sativa *and *Populus trichocarpa *for comparative analysis and knowledge integration. BLASTX results with the *Arabidopsis *proteome were used for the assignments of curated Gene Ontology terms and pathways from TAIR [21] for each annotated GSS. BLASTX results with UniProtKB-Swiss-Prot were used for data integration with the ENZYME database [25].

### Homology-based annotation

Searches were performed with each cowpea GSS using BLASTX with cutoff expectation (e) value of 1e-8, against UniProtKB-TrEMBL [26], UniprotKB-Swiss-Prot [27], NCBI GenBank Proteins [28], and UniProtKB-PIR (Protein Information Resource) [29] public FASTA formatted protein databases.

### HMM-based gene-modeling and domain finding

The potential domains on annotated GSS were analyzed using the HMMER package [30] against the Pfam database [31,32]. Possible exons and introns in each cowpea GSS were predicted using the HMM-based Genscan gene predication program [33,34]. Although Genscan has not been widely applied to plant systems, we found that it in combination with the use of the HMMER package gives a reasonable estimate of coding potential. Additional gene predictions programs, such as FgeneSH that are better optimized for use in plant systems, are being applied and the results will be added to the database as they become available.

### Tandem repeat finding

GGS data from cowpea should be an invaluable resource for the development of molecular markers and genetic maps for comparing syntenic relationships among legume and non-legume species. Simple sequence repeat (SSR) markers are one of the popular DNA markers for plant genome analysis and marker-assisted selection in crop breeding programs. Traditionally, SSR markers were generated through screening of SSR-enriched genomic libraries, a process that was very time-consuming and expensive. Recently, *in-silico *methods have been developed that allow rapid discovery of potential SSR markers from plant DNA sequence (EST, genomic fragments, BAC ends) datasets. Each GSS was analyzed using the Tandem Repeats Finder program [33] developed by Benson [34]. The identity of the GSS containing one or more SSRs, along with information on repeat size, composition, and the primers for their amplification were parsed and loaded into relational tables for sorting, search, and joining.

## Utility

**Figure 2:**
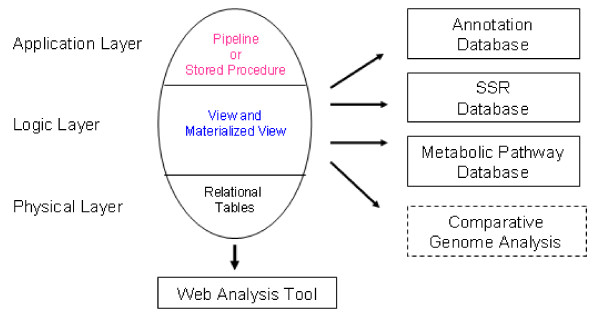
The relational database architecture of the data management system for cowpea methylation filtered genomic genespace sequences.

We used the PostgreSQL [35] relational database management system to manage and organize sequence information and to disseminate the results of our analyses. The database was implemented in a layered approach, with relational tables in key-value pair fashion as the physical data storage layer for raw data and analysis results management; views and materialized views as logical layer for the representation of biological knowledge derived for the integration of processed data, and analysis pipelines and database stored procedures as the application layer for data update, retrieval, curation, and validation. The materialized views were indexed on each attribute for quick data access, table join, and knowledge retrieval. The three knowledge bases (i.e., SSR database, metabolic pathway database, and annotation database) were implemented at the logical layer and curated, validated, and updated at the application layer based on the data stored in the physical relational tables (see figure 2). The development and test database instance is hosted on  and the production instance is located on . Both instances are synchronized with the additions of newly processed results, such as contig building, contig annotation and comparative analysis with legume unigenes.

We have also created an easy-to-use interface for the knowledge bases, mostly based on CGI written in Perl running on an Apache web server [36]. Our user interface has several components. The most important ones are data download, sequence and library statistics, analysis toolkit, and the SSR, metabolic pathway, and annotation knowledge bases.

### Cowpea GSS Knowledge bases

#### (1) The cowpea GSS annotation database

**Figure 3:**
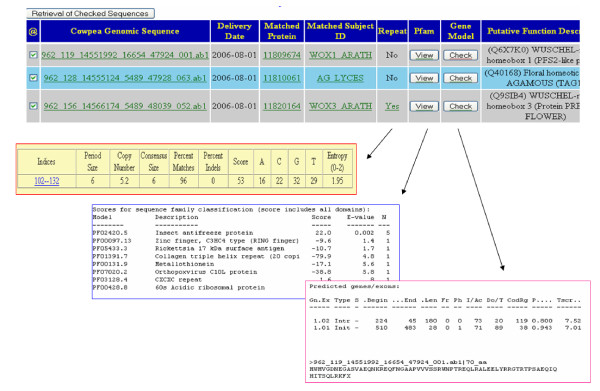
Snapshots of annotation database based on cowpea methylation filtered genomic genespace sequences.

We combined the web interface of the annotation with the comparative plant proteome analysis from the genome sequence-derived proteomes of *Medicago truncatula, Arabidopsis thaliana*, *Oryza sativa *and *Populus trichocarpa*. The interface was designed for queries via UniProtKB accession number, GenBank GI number, GSS sequence identification number, sequence feature on annotation, and ortholog/homolog identified via blast homology-based sequence alignments against the above four public protein sequence databases (see figure 3).

Between 13 and 31% of the total GSS were annotated by homology-based annotation against these publicly available protein knowledge bases and the four plant proteomes (see table 1). When all of the distinct annotated GSS are combined, approximately 36% of the total cowpea GSS could be assigned putative functions by comparison to the publicly available annotation protein datasets. Studies underway aimed at estimating the robustness of our dataset indicate that we tagged approximately 40,000 gene coding regions representing between 19,786 and 23,561 unique GenBank Accession numbers (Timko, M.P., manuscript in preparation). Knowledge integration was done at both database level through relational table joining (for SSR, Pfam domain annotation, gene model, putative functional assignment) and interface level through http hyperlinks (for linking with knowledge sets at PIR, UniProtKB, NCBI, TAIR, and TIGR).

**Table 1:** Annotation results with a total of 263,425 GSS via a homology-based approach

Annotation Databases	Annotated Cowpea GSS	Distinct Accession Numbers	Annotation Database Size	Percentage of Matched Sequences
NCBI GenePeptide	78,787	23,561	3,440,254	29.91
UniProtKB PIR	67,807	12,921	283,416	25.74
UniprotKB Swiss-Prot	34,738	6,676	211,104	13.19
UniProtKB TrEMBL	78,102	23,031	2,638,494	29.65
*Arabidopsis thaliana*	77,591	14,561	25,920	29.46
*Oryza sativa*	69,993	15,708	62,826	26.57
*Medicago truncatula*	61,711	7, 406	24,420	23.43
*Populus trichocarpa*	82,957	19,868	45,555	31.49

Annotation results with a total of 263,425 GSS.

#### (2) The cowpea SSR database

The interface allows users to query the database by cowpea GSS ID, consensus pattern, repeat copy number, consensus size, or richness of nucleotides in the repeats. A total of 30,877 SSRs were identified among the GSS, with 3,717 SSRs located in GGS with homology to known genes. All identified SSRs are available for downloading from the CGKB.

#### (3) The cowpea metabolic pathway database

**Figure 4:**
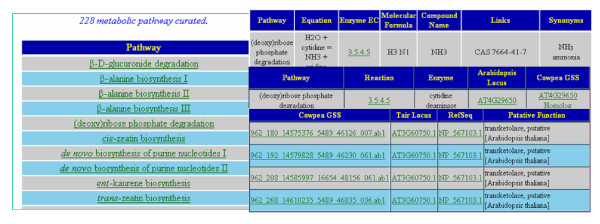
Snapshots of the metabolic pathway database based on cowpea methylation filtered genomic genespace sequences.

Data integration for the metabolic pathways database was performed using the TAIR metabolic pathway knowledge datasets for both *Arabidopsis *and cowpea orthologs and homologs. Data integration for enzymes was performed using both UniProtKB-Swiss-Prot and UniProtKB-TrEMBL. The web interface can be queried for 228 curated plant metabolic pathways in TAIR and for individual enzymes using the cowpea sequence identification number, UniProtKB access number, GenBank GI number, enzyme EC number, enzyme or cofactor name, and key word search on putative assigned functions (see figure 4).

### The web-based sequence analysis toolkit

We also provided several web-based data analysis programs for users to perform data mining activities. The tools are focused on quick sequence retrieval and contig building of cowpea GSS. Below is a brief description of the programs.

(1) BlastAll is a local installation of NCBI BLAST program with the cowpea GSS FASTA formatted sequence database. This tool can be used to retrieve homologous overlapping cowpea sequences for sequence extension and contig building.

(2) RetrieveAll is a program for quick cowpea GSS sequence retrieval via sequence ID or trace name.

(3) Contig Builder is a local web based implementation of the Phrap program for contig building. Overlapping or homologous cowpea GSS can be uploaded into this tool to extend the sequence length or make contigs based on sequence overlaps.

(4) Multiple Sequence alignment is a local web based implementation of the CLUSTALW multiple sequence alignment program to check the quality of the overlapping GSS region for forming contigs or extending the length of GSS sequences.

## Discussion and conclusion

Genome-related public databases are an invaluable part of the scientific community. There are two major users of these resources. The first is the scientific focus group actively studying the target system or organism. Among this target audience are breeders who can use this resource for the design of molecular markers for use in marker-assisted breeding and introgression programs in cowpea and other legumes. The second group is the broader scientific community interested in relating this specialized information to other systems/organisms. The aim of the CGKB is to provide an annotated, well-organized, and rigorously analyzed dataset of MF clone sequences as a resource for cowpea researchers and pan-legume crop specialists. We have found that comparisons to the NCBI GenBank Protein and UniProtKB-TrEMBL allow for the best coding potential detection of the cowpea GSS since these protein knowledgebases represent global collection of proteins. The UniProtKB-Swiss-Prot is mainly useful for data integration of known domains and enzyme databases. Comparison of the cowpea GSS to the *Arabidopsis thaliana *proteome provides for comparative genome analysis and integration with plant related GO terms and metabolic pathways from TAIR.

The structure and organization of the CGKB allows for rapid modification of data storage and retrieval and addition/removal of functionalities. Among the future plans for the database are (i) contig building and singlet estimation on the cowpea genomic genespace sequences; (ii) incorporation of PCR primer sequence information for SSR amplification into the SSR database and other data analysis tools associated with marker development for cowpea; (iii) integration with cowpea genetic mapping activities for identification of potential trait-linked markers; (iv) BLASTX analysis with the *Medicago *proteome will be used to anchor cowpea GSS to the physical contig and genetic map of *Medicago truncatula *and inclusion of additional comparative genomic analysis and syntenic relationships to other legume and non-legume species; and (v) full data integration at database physical level with *Arabidopsis *and rice knowledge bases.

## Availability and requirements

The CGKB is publicly available at the URL  The CGKB is published under the GNU General Public License (GPL) which implements the understandings of the Kirkhouse Trust Intellectual Property Statement.

We have chosen the GPL as the best way to ensure free and unrestricted access to the cowpea genomic data, and to subsequent discoveries resulting from use of this data. This free exchange of knowledge benefits the poor farmers of the world and promotes rapid scientific progress. Users are asked to register at the CGKB site: 

## Authors' contributions

TWL participated in the design of the CGKB database and database system administration. XC performed the bioinformatics data analysis and web implementation. PJR contributed the web tool design, validation, and data analysis. TAS contributed project coordination, computer cluster setting up and administration. MPT performed data and web interface validation and served as the principal investigator of the project. All authors have assisted in the writing and have read and approved the final manuscript.
